# Impact of the Empower-DSD training program on the health-related quality of life in children, adolescents and young adults with differences of sex development and their parents

**DOI:** 10.3389/fped.2026.1719768

**Published:** 2026-07-30

**Authors:** Uta Neumann, Ralph Schilling, Sabine Wiegmann, Martina Ernst, Mirja Winter, Katja Wechsung, Louise Marshall, Ulla Döhnert, Olaf Hiort, Julia Rohayem, Klaus-Peter Liesenkötter, Annette Richter-Unruh, Gerda Janssen-Schmidchen, Gloria Herrmann, Martin Wabitsch, Stephanie Roll, Thomas Keil, Barbara Stöckigt

**Affiliations:** 1 Clinic for Pediatric Endocrinology and Diabetology, Centre for Chronic Sick Children, ENDO-ERN-Centre, German Centre for Child and Adolescent Health (DZKJ)-Partner Site Berlin, Charité – Universitätsmedizin Berlin, Berlin, Germany; 2 Institute of Social Medicine, Epidemiology and Health Economics, Charité – Universitätsmedizin Berlin, Berlin, Germany; 3 Division of Pediatric Endocrinology and Diabetes, Department of Pediatrics and Adolescent Medicine, University of Luebeck, Luebeck, Germany; 4 Centre for Reproductive Medicine and Andrology, Clinical and Operative Andrology, University Hospital Münster, Münster, Germany; 5 Ostschweizer Kinderspital, Endocrinology, St. Gallen, Switzerland; 6 Centre for Hormonal and Metabolic Disorders, Endokrinologikum Berlin, Berlin, Germany; 7 MVZ Dr. Eberhard & Partner Dortmund, Dortmund, Germany; 8 SHG Interfamilien, c/o Paritätischer Wohlfahrtsverband Niedersachsen e.V., Kreisverband Emden, Emden, Germany; 9 Centre for Rare Endocrine Diseases (ZSEE), Division of Pediatric Endocrinology and Diabetes, Department of Pediatrics and Adolescent Medicine, German Centre for Child and Adolescent Health (DZKJ)- Partner Site Ulm, Ulm University Medical Centre, Ulm, Germany; 10 Institute for Biostatistics and Informatics in Medicine and Aging Research, University Medical Center Rostock, Rostock, Germany; 11 Institute of Clinical Epidemiology and Biometry, University of Würzburg, Würzburg, Germany; 12 State Institute of Health I, Bavarian Health and Food Safety Authority, Erlangen, Germany; 13 Institute of General Practice and Interprofessional Care, University Hospital Tuebingen, Tuebingen, Germany; 14 Robert Bosch Center for Integrative Medicine and Health, Bosch Health Campus, Stuttgart, Germany

**Keywords:** adolescents, children, differences of sex development (DSD), health related quality of life (HRQoL), multidisciplinary training programme, acquire diagnosis-specific knowledge

## Abstract

**Background:**

Treatment of individuals with a difference of sex development (DSD) must respect ethical principles, human rights, and national laws. Informed consent is central and should be supported by age-appropriate information from early on. In Germany, genital surgery in DSD is prohibited until autonomous consent is possible, requiring sufficient maturity and proper information. DSD may negatively affect individuals and their families' health-related quality of life (HRQoL) and may be associated with stigmatization, inadequate information and lack of peer counseling. We examined if HRQoL of affected individuals and their parents can be improved by a newly developed multidisciplinary training program.

**Methods:**

In collaboration with self-help organisations, the Empower-DSD consortium developed and implemented a two-day modular educational program for children and young adults (6–24 years) with DSD (Congenital adrenal hyperplasia, Turner-syndrome, Klinefelter-syndrome, other 46,XX and 46,XY-DSD diagnoses) and their families, addressing diagnosis-specific and psychosocial topics. From 08/2020 to 09/2022, 104 multidisciplinary sessions were conducted in five German DSD centres. As primary endpoint, participants completed HRQoL questionnaires (WHO-5, KINDL) at baseline, and three and six months post-program. Data were analyzed separately for children (6–13), adolescents (14–17), young adults (18–24), and parents. Additionally, qualitative interviews and participatory observations of children's sessions were analyzed using qualitative content analysis within a mixed methods approach.

**Results:**

We evaluated 102 children, 95 adolescents, 56 young adults, and 380 parents/relatives. Questionnaire completion rates were 96% at baseline and 60% at 6 months. HRQoL was stable or improved in over 66% of children and parents, and in 57% of adolescents and young adults at 6 months. Qualitative analysis of 71 interviews and four observed sessions indicated participants felt empowered by the psychological support and the exchange with peers and valued the opportunity to discuss their diagnosis openly.

**Conclusion:**

Children with DSD and their parents seemed to benefit more from the new education program than adolescents and young adults with DSD. Overall, the two-day program encourages engagement with DSD diagnosis. The exchange with other people with DSD or parents was experienced as empowerment. Further research should include long-term follow-ups to determine the sustainable effects of multidisciplinary training programs.

**Clinical Trial Registration:**

https://innovationsfonds.g-ba.de/projekte/empower-dsd.219, identifier DRKS00023096.

## Introduction

Differences of sex development (DSD) are classified according to karyotype into chromosomal DSD, 46,XX-DSD and 46,XY-DSD (1). The different diagnoses can affect gonadal function, development of internal and external genitalia, sex hormone synthesis or action and fertility. DSD are rare, often genetically determined conditions and clinical signs can appear at different stages of life, for example with intersex genitalia at birth, with delayed pubertal development or with infertility later in life (1). In addition to its impact on physical development, a DSD diagnosis in children is associated with reduced health-related quality of life (HRQoL), particularly affecting self-esteem, physical well-being, and school performance (2).

HRQoL is a concept that includes physical and mental health perceptions and their correlates, including health risks and conditions, functional status, social support, and socioeconomic status. Assessing HRQoL is considered a valid indicator of intervention outcomes and can examine the burden of the diagnosis (3). In cases where symptoms are primarily somatic, such as asthma, and effective treatment is available, the burden is likely to be reduced and the impact on HRQoL minimal (4). The situation is different for diagnoses with highly stigmatizing, isolating, and demotivating components, such as obesity or attention deficit hyperactivity disorder (ADHD), where there are clear correlations with lower levels of protective factors and HRQoL (4). DSD diagnoses can also be stigmatizing and shameful as they can affect the development of sexual characteristics, gender identity and psychosexual maturation.

Until the end of the 1990s, the care of people with DSD was characterized by the so-called “optimal gender policy” (5). Physicians often decided on the assigned gender of the child and the children themselves remained unaware about their diagnosis (6). People with DSD often perceived secrecy surrounding their diagnosis and its treatment (7). In addition, age-appropriate medical information is often lacking and peer counseling is not available to all those affected (8). This could lead to the person's own identity not matching their social gender, without them being able to find an explanation for this (9). It is largely due to the work of people with DSD that the care and treatment approach for people with DSD has changed to one based on informed decision-making involving the individual. The German Ethics Council has drawn up recommendations for the care of intersex people in dialogue with the people with DSD themselves and the respective self-help organization (9). Important points are, for example, care by an interdisciplinary team specialized in DSD, contact with peer counseling and extensive counseling for the person diagnosed with DSD and, in childhood, also for their parents. Decisions on the use of irreversible sex reassignment surgery should only be made once the person is able to give consent. This means that measures that are not absolutely necessary should be postponed until children or young people are old enough to make their own decisions. This resulted in the German Act on the Protection of Children with DSD, which was passed in 2021. It states that medical procedures on children who are unable to give consent are prohibited if they are performed for the sole purpose of assigning sex (10). However, these restrictions, which were put in place to protect children who are unable to give consent, pose major challenges for parents of new-borns and young children diagnosed with DSD, particularly at the time of diagnosis. Parents' concerns relate to many areas of life and questions can raise about physical development, gender identity and fertility, as well as life aspects such as sex assignment on the birth certificate, gender of upbringing or the selection of first name. Comprehensive information about the diagnosis, diagnostic measures and treatment options and their effects and side effects is necessary for informed decision-making by people with DSD and, in the case of children, their parents (7, 11), such as naming, openness about the diagnosis to others, health risks, stigma, fertility and the possibility of a self-determined and happy life. A holistic approach to treating children with a chronic condition such as DSD includes the consideration of the impact on the individual and its family, and identifying coping resources and strategies (4). An age-appropriate education to establish the possibility for informed consent in people with DSD should be integrated into the routine care (12). This was addressed in the project Empower-DSD financed by the Innovation fund of the German statutory health insurance companies from 2019 to 2023. Modular training programs were developed for the diagnoses congenital adrenal hyperplasia (CAH), Turner-syndrome (TS), Klinefelter-syndrome (KS) and one for the remaining DSD-diagnoses [XX-DSD, XY-DSD, chromosomal DSD/mosaicism, Mayer-Rokitansky-Küster-Hauser syndrome (MRKH)—summarized as XX-/XY-DSD] (13). The impact of participation in the training program on participants' HRQoL and life satisfaction was evaluated using a mixed-methods approach involving quantitative and qualitative methods.

### Patient and stakeholder involvement

The Empower-DSD program has been developed in a unique participatory approach by involving different medical and psychologic specialists and the German DSD self-help organizations representing the included diagnoses CAH (AGS – Eltern- und Patienteninitiative e.V.), XX-/XY-DSD [Intergeschlechtliche Menschen e.V. (IMeV), SHG Interfamilien], Klinefelter syndrome (47xxy klinefelter syndrom e.V.), and Turner syndrome (Turner-Syndrom Vereinigung Deutschland e.V.). The participation of the self-help organizations and inclusion of their valuable knowledge and experience during the whole Empower-DSD project supported the development of a needs-based program (13, 14), and enabled a qualified and professional peer counseling during the training sessions in each target and age group. Often, tandem peer counseling was available to present the different perspectives of the parents, and the young adults with a DSD diagnosis.

## Methods

### Study design for quantitative evaluation

A detailed description of the study procedure has already been published (13). Children, adolescents and young adults aged 6–24 years with a confirmed diagnosis of CAH, KS, TS or one of the remaining DSD-diagnoses according to the Chicago-classification (1), summed up as XX-/XY-DSD (including other chromosomal DSD, 46,XX-DSD, 46,XY-DSD, MRKH) and their parents/relatives were informed about the training program during their visit in the outpatient clinic or through the DSD self-help organization in Germany. A total of 102 children (6–13 years), 95 adolescents (14–17 years), 56 young adults (18–24 years) with a DSD diagnosis and 380 parents were included in the study. Between July 2020 and September 2022, they took part in the EMPOWER-DSD training program, which was offered in five German centres with DSD expertise. The training courses were delivered in face-to-face group sessions. For the evaluation, all participants were asked to complete an online questionnaire before (baseline) and three and six months after completing the program. HRQoL was assessed by self-report questionnaires using KINDL for children and adolescents (15–17), which ranges from 0 (= worst) to a maximum of 100 points (= best), and WHO-5 (total score scaled to a range of 0–100 points; the higher the score, the better the HRQoL) for young adults, parents and relatives (18). Further parameters assessed included body image (Body image scale – BIS, ranging from 0 = worst to a maximum of 100 points=best), age (in years), diagnosis (in four groups, CAH, KS, TS and Other DSD), educational level [only in young adults and parents, divided in three categories high, medium, and low according to ISCED (19)].

### Statistical methods

Descriptive statistical analyses were conducted for children, adolescents, young adults and parents/relatives (*n* = 6 relatives included). Study participants with a DSD diagnosis were aged 8–24 years. For CAH, children aged 6 and 7 years were also included if they were developmentally ready. The group of parents/relatives was split into two groups: one consisting of parents and relatives whose children had been diagnosed with DSD within the last two years before recruitment, and the other consisting of parents/relatives of children with longstanding diagnoses. All parents/relatives who had consented to participate and attended at least one day of training were evaluated. For categorial variables we utilized absolute and relative frequencies, whereas for metric variables minimum, maximum, mean standard deviation (SD), median and interquartile range were calculated. According to Gong et al. minimal important differences (MID) for children and adolescents were defined by a difference of 5% of the total achievable score after three and six months compared to baseline and by a difference of 10% for young adults and parents (20). No adjustment for multiple testing was applied in this exploratory study. Statistical calculations were performed using IBM SPSS Statistics for Windows, Version 27.0. Diagrams were created using Microsoft Excel 2019 MSO (16.0.10416.20027) and Microsoft PowerPoint (version 2019) MSO (16.0.10416.20027).

### Multivariable analysis in children and adolescents

To identify predictors for HRQoL of children and adolescents, we carried out a multivariable analysis using multiple linear regression. The outcome HRQoL in this age group was assessed at three and six months after the training program by the total KINDL score. Potential predictors included in the modelling were the diagnosis and age of the child, the total score of BIS, and the parents' HRQoL. Diagnosis, age and educational level were only measured at baseline. Furthermore, in the analyses, we adjusted for the participants HRQoL at baseline. Missing data were imputed using multivariate imputation by chained equations based on 30 imputed data sets and predictive mean matching. 30 complete datasets were created and analyzed separately. The results were then combined using Rubin's rules (21).

### Multivariable analysis in parents

To identify factors associated with the parents' HRQoL, a regression was calculated using a linear mixed effects model. Parent/child dyads were formed by linking the data from parents and their own children. The total WHO-5 score at three and six months was used as the outcome variable for the parents' HRQoL. Initially, the child´s diagnosis, the age of the parents, the educational level and the parents' WHO-5 score at baseline were included in the modelling as fixed effects; the children's total HRQoL score at the respective measurement time of the outcome was included as a random effect. The quality of the model was determined using Akaike's (AIC) and Schwarz's Bayesian criterion (BIC). Finally, the age, educational level and parental HRQoL at baseline were included in the modelling as fixed effects.

### Qualitative evaluation and analysis

The semi-structured interviews were planned with individuals with DSD in the different age groups and their parents/relatives, peer counsellors and professionals from the study centres. To ensure that the interview guidelines will cover all relevant aspects regarding the completeness and accuracy of the Empower-DSD program, the interview guidelines were developed in a participatory manner. That means, it was developed on the basis of knowledge and experiences of all stakeholders like physicians, psychologists, social workers, as well as the representatives of the different support groups (persons with DSD itself and parents). Therefore, the developer of the guidelines took part in the different group and project meetings, train-the-trainer-academies, personal interviews with stakeholders and considered also expert articles, daily press information and blogs of persons with DSD (14). On the basis of these interview guidelines, the interviews were conducted individually (e.g., with professionals, peers) or as multi-person interviews, for example minors with DSD and their parents. The interviews were scheduled as face-to-face or telephone interviews in all centres. Telephone interviews were prioritized due to the COVID-19 pandemic. The interviews took place during the whole study period in peers and professionals, but only after participation in the training program in people with DSD and their parents. All interviews were completed in German and if quotes were included in the manuscript, they were translated into English. The translation was done by the interviewing person.

The qualitative interviews focussed on the evaluation of the training program regarding: expectations, wishes, experiences, impact on handling with the diagnosis, shared-decision-making and in professionals and peers additionally regarding the influence on job satisfaction. Professionals who delivered the training or have been involved in its development and peers, who participated in the training as peer counsellors or who have been involved in the development of the training were included. As this method is not feasible in children without any basis of trust between the interviewer and interviewee, particularly in the case of children and adolescents, the methodology was supplemented by participatory observations of training sessions in one centre. The interviews were digitally recorded, pseudonymously transcribed and gradually coded, categorized and analyzed on the basis of a qualitative content analysis (8). Observation protocols were written about the observed situations within the training courses and then coded, categorized and analyzed step by step. The coding was carried out inductively (from the data material) and deductively (according to the research question and the interview guide). The data was analyzed using MAXQDA® software. As one supplementary method, a word cloud was used for analysis to visualize qualitative data (22).

### Triangulation

Both quantitative and qualitative data were collected in parallel. Following the mixed methods approach of this study, quantitative and qualitative findings were triangulated based on both data and results. Thus, results were expanded, enlarged and deepened (23). The qualitative analyses of the interviews with individuals with DSD and their parents were triangulated with quantitative findings on HRQoL.

This study has been prospectively registered in the German Clinical Trials Register (DRKS) under the identifier DRKS00023096.

## Results

### Quantitative analysis

A total of 633 participants of the training courses were included in the evaluation. A detailed description of the groups and participants is given in Table 1, further details were published by Wiegmann et al. (24).

**Table 1:** Baseline age, health-related quality of life (HRQoL) and body image of EMPOWER-DSD study participants, by age group.

Characteristics	Children (6–13 y.)	Adolescents (14–17 y.)	Young adults (18–24 y.)	Parents of newly diagnosed children^a^	Parents of children with longstanding diagnosis^b^
Age in years. mean (SD)	10.6 (1.9)	15.2 (1.0)	19.6 (1.9)	41.1 (9.3)	47.0 (8.1)
n (missing)	102 (0)	95 (0)	56 (0)	136 (3)	228 (13)
Diagnosis			5 (8.9)		
CAH, *n* (%)	31 (30.4)	15 (15.8)	17 (30.4)	38 (27.3)	57 (23.7)
KS, *n* (%)	17 (16.7)	43 (45.3)	8 (14.3)	30 (21.6)	78 (32.4)
TS, *n* (%)	33 (32.4)	19 (20.0)	26 (46.4)	20 (14.4)	57 (23.7)
Other DSD, *n* (%)	21 (20.6)	18 (18.9)		51 (36.7)	43 (18.8)
HRQoL					
KINDL mean (SD)	74.3 (9.9)	56.2 (9.0)			
Median (Q1|Q3)	77.1 (65.6|82.3)	55.7 (48.8|62.5)			
n (missing)	95 (7)	65 (30)			
WHO-5 mean (SD)			57.2 (19.3)	56.7 (20.3)	60.6 (18.2)
Median (Q1|Q3)			56 (44|71)	64 (44|72)	64 (52|72)
n (missing)			48 (8)	123 (16)	217 (24)
Body image scale					
mean (SD)	60.5 (20.7)	65.9 (21.9)	64.1 (19.6)		
Median (Q1|Q3)	62 (36|73)	69 (50.3|80)	65 (48.5|78.5)		
n (missing)	27 (75)	64 (31)	37 (19)		

a Diagnosed within the last 2 years.

b including parents and relatives.

### HRQoL in children, adolescents, young adults and parents

Overall, participants of the Empower-DSD training program showed stable HRQoL scores (measured by KINDL-questionnaire in children and adolescents (Tables 2, 3) and by WHO-5 in young adults and parents/relatives) (Table 4). Six months after participating in the Empower-DSD training program, parents of newly diagnosed children with DSD showed a slight increase in HRQoL, while HRQoL in parents of children with longstanding diagnosis remained constant (Table 4).

**Table 2:** KINDL (health-related quality of life) and six subdimensions in children aged 6–13 years at baseline, 3 and 6 months.

	At baseline		At 3 months		At 6 months	
KINDL-score	Mean (SD)	n (missing)	Mean (SD)	n (missing)	Mean (SD)	n (missing)
Total score	74.3 (9.9)	95 (7)	73.4 (10.1)	70 (32)	73.6 (9.0)	55 (47)
Physical wellbeing	74.2 (17.4)	94 (8)	72.1 (16.2)	71 (31)	74.9 (14.5)	94 (8)
Psychological wellbeing	77.5 (13.2)	94 (8)	76.6 (12.8)	71 (31)	75.6 (10.9)	55 (47)
Self-esteem	60.3 (18.5)	94 (8)	61.2 (17.2)	71 (31)	62.3 (15.3)	55 (47)
Family	81.9 (12.6)	95 (7)	80.9 (14.9)	71 (31)	82.6 (13.6)	55 (47)
Friends	76.1 (14.3)	95 (7)	74.5 (14.8)	69 (33)	71.2 (16.1)	54 (48)
School	76.1 (16.6)	93 (9)	76.0 (15.4)	69 (33)	74.5 (16.0)	55 (7)

**Table 3:** KINDL – (health-related quality of life) and six subdimensions in adolescents aged 14–17 years at baseline, 3 and 6 months.

	At baseline		At 3 months		At 6 months	
KINDL-score	Mean (SD)	n (missing)	Mean (SD)	n (missing)	Mean (SD)	n (missing)
Total score	56.2 (9.0)	65 (30)	56.0 (9.3)	51 (44)	57.8 (10.6)	39 (56)
Physical wellbeing	64.0 (15.4)	44 (51)	64.3 (14.5)	46 (49)	67.7 (13.5)	29 (66)
Psychological wellbeing	71.5 (12.2)	53 (42)	70.2 (13.8)	38 (57)	74.1 (13.1)	27 (68)
Self-esteem	40.3 (17.3)	85 (10)	40.8 (18.8)	61 (34)	41.5 (18.6)	47 (48)
Family	66.0 (13.4)	48 (47)	64.1 (15.1)	41 (54)	66.5 (13.4)	25 (70)
Friends	54.2 (15.1)	80 (15)	48.1 (15.2)	63 (32)	52.8 (15.4)	44 (51)
School	55.5 (15.6)	73 (22)	56.5 (13.3)	56 (39)	58.6 (15.3)	43 (52)

**Table 4:** WHO-5 (health-related quality of life) in young adults aged 18–24 years and parents/relatives of a child with DSD at baseline, 3 and 6 months (mean, 95% confidence interval).

	At baseline		At 3 months		At 6 months	
WHO-5-score	Mean (SD)	n (missing)	Mean (SD)	n (missing)	Mean (SD)	n (missing)
Young Adults	57.2 (19.3)	48 (8)	60.4 (19.9)	37 (19)	55.4 (18.3)	28 (28)
Parents of newly diagnosed children	56.7 (20.3)	123 (16)	61.2 (19.7)	100 (39)	62.6 (14.5)	77 (62)
Par. of children with longstanding diagnosis	60.6 (18.2)	217 (24)	61.3 (20.2)	162 (79)	61.0 (20.1)	128 (113)

When only those participants who completed the questionnaires at all three assessment points were considered, the HRQoL of participants with DSD showed a similar trend to that observed when all participants were included [Mean (SD)] at baseline, 3 months and 6 months in children [73.8 (10.2); 73.4 (10.4); 73.5 (9.1); *n* = 54], young adults [55.7 (18.8); 59.4 (19.6); 55.0 (18.5); *n* = 27] and parents of newly diagnosed children [59.3 (17.7); 62.0 (18.6); 62.4 (18.7); *n* = 69], while adolescents [58.8 (9.3); 56.3 (9.6); 55.5 (11.2); *n* = 27] and other parents [61.7 (18.8); 59.2 (21.3); 60.8 (20.4); *n* = 118] showed a slight decrease of HRQoL at 6 months.

**Figure 1:**
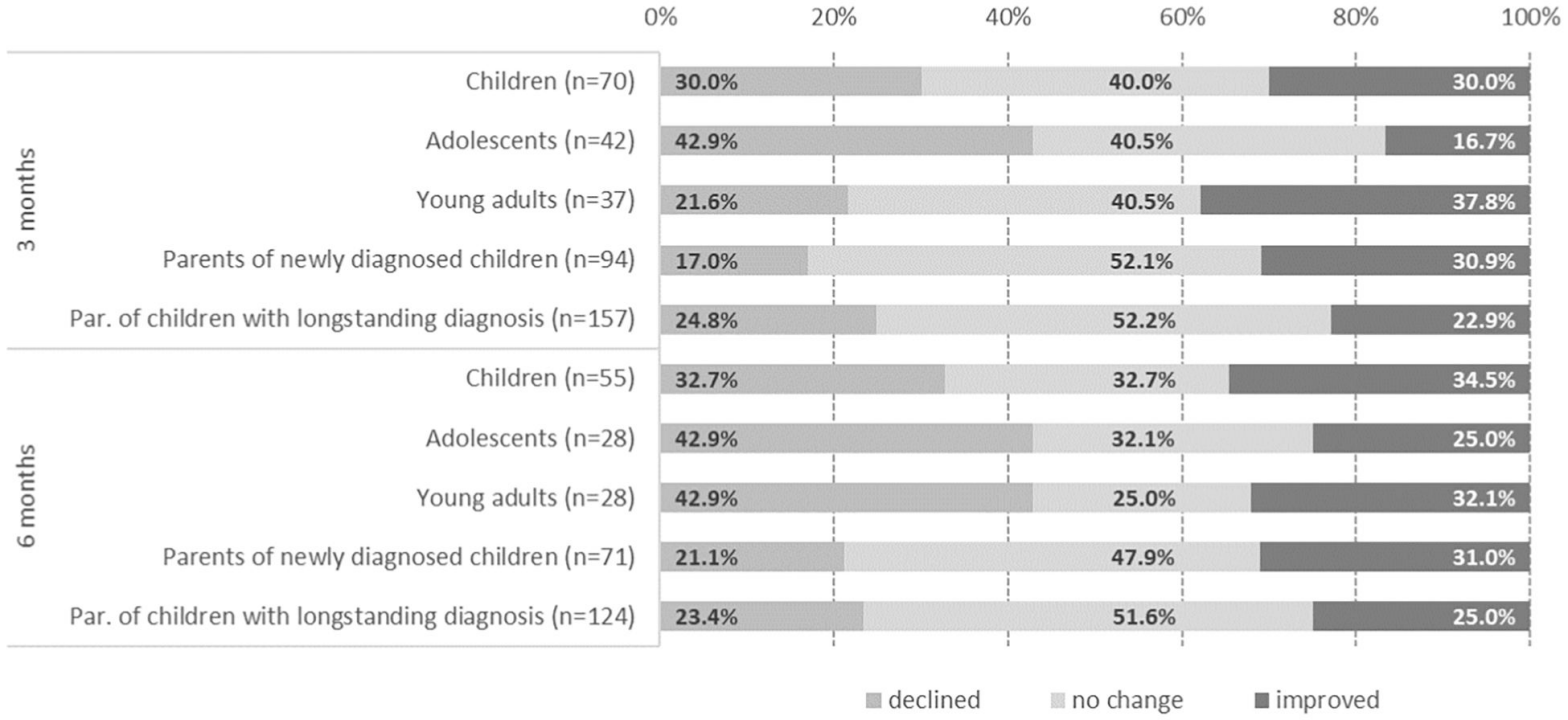
Relevant change in HRQoL according to defined MID (defined by a difference to the baseline score of 5% in children and adolescents and 10% in young adults and parents). HRQoL was analyzed using the questionnaires KINDL (children, adolescents) and WHO-5 (young adults, parents/relatives).

However, individual differences are apparent when looking at the relevant changes from baseline. Three months after participating in the Empower-DSD program, 30.0% of children, 17% of adolescents and 38% of young adults showed a relevant improvement in HRQoL. At 6 months, these proportions were even higher in children (35%) and adolescents (25%), while the proportion decreased by about 6 percentage points to 32% in young adults. The proportion of parents with relevant improvements in HRQoL ranged from 23% to 31% at 3 months and 25% to 31% at 6 months after the training. On the other hand, a proportion of participants also experienced a deterioration in their HRQoL after 3 months, particularly adolescents (30% at 3 months, 42.9% at 6 months) and young adults (42.9% at 6 months) (Figure 1).

### KINDL and KINDL-subdimensions

At baseline the lowest scores were detected in the categories self-esteem, friends and school, especially for adolescents, while the children's scores were mainly reduced in the category self-esteem. After 3 and 6 months, all sub-dimensions showed slight improvements in adolescents, while in children all but the sub-dimensions friends and school slightly increased. In both groups, self-esteem had the lowest scores of all sub-dimensions (Tables 2, 3).

### Body image scale

**Figure 2:**
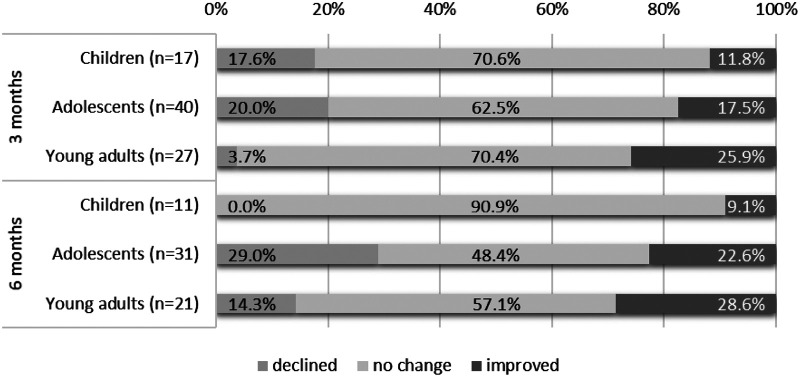
Difference in Body image scale from 6 months to baseline in children (A), adolescents (B) and young adults (C) who participated in the Empower-DSD program.

Baseline values for body image of children, adolescents and young adults are shown in Table 1. During the course of the study, slight changes in mean body image were observed for all groups (children at 3 months: 61.4 ± 25.2 (*n* = 21), 6 months: 63.2 ± 20.1 (*n* = 13); adolescents at 3 months: 65.2 ± 25.5 (*n* = 52), 6 months: 58.4 ± 24.1 (*n* = 42); young adults at 3 months: 67.9 ± 22.0 (*n* = 33), 6 months: 71.2 ± 23.6 (*n* = 23). Participation in the training program resulted in a clinically relevant improvement especially in young adults (Figure 2).

### Multivariable analysis in children and adolescents

A total of 156 children and adolescents were included in the multivariable analysis. At three months, a lower HRQoL was associated with poorer body image, increasing age of the children and a lower HRQoL at baseline. These associations remained similar at six months except for body image (Table 5).

**Table 5:** Predictors of HRQoL of children and adolescents (according to KINDL total score).

		After 3 months			After 6 months	
Predictor	b	SE	95%-CI	b	SE	95%-CI
Diagnosis	0.328	1.130	−1.91–2.57	0.679	1.697	−2.72–4.08
HRQoL of parents^a^	0.065	0.055	−0.04–0.17	0.039	0.079	−0.12–0.20
HRQoL at baseline	0.537	0.109	0.32–0.75	0.419	0.130	0.16–0.68
Body Image	−0.135	0.065	−0.27 to −0,004	−0.080	0.056	−0.19–0.03
Age of participant	−1.117	0.473	−2.05 to −0.18	−1.351	0.517	−2.37 to −0.33

a WHO-5 Total score transformed into 0–100 scale.

b-regression coefficient, SE-standard error, 95% confidence interval.

### Multivariable analysis of HRQoL in parents

The multivariable analysis of predictors for the parental HRQoL included only the parent-child dyads (*n* = 111 at 3 months, *n* = 77 at 6 months). After 3 and 6 months, the children's HRQoL measured at these time points and the parental HRQoL at baseline were associated with the parent's HRQoL, whereas no associations with the parent's age or educational level were found (Table 6).

**Table 6:** Predictors of parental HRQoL (according to wHO-5 total score).

	At 3 months			At 6 months		
Predictor	b	SE	95%-CI	b	SE	95%-CI
Educational level						
low	2.527	9.068	−15.52–20.58	9.574	17.106	−24.52–43.67
middle	1.379	4.541	−7.64–10.39	−8.897	5.001	−18.88–1.09
high (ref.)	-	-	-	-	-	-
Age of parents	0.072	0.241	−0.41–0.55	0.170	0.290	−0.41–0.75
WHO-5 Score at t0	0.664	0.849	0.50–0.83	0.486	0.093	0.30–0.67
HRQoL of children^a^	0.310	0.131	0.05–0.57	0.400	0.161	0.08–0.72

a HRQoL of children after 3 months and after 6 months, model quality at 3 months (AIC 954.595, BIC 976.271) and at t3 (AIC 657.286, BIC 676.036).

b-regression coefficient, SE-standard error, 95% confidence interval.

### Results of qualitative analysis

The qualitative evaluation included the analysis of 71 individual interviews: 19 interviews with children, adolescents and young adults with a DSD-diagnoses (Table 7) and additional interviews with 17 parents, 16 peers, 19 professionals and 4 participant observations of training sessions including two for XX-/XY-DSD, 1 for CAH and 1 for TS.

**Table 7:** Number of interview partners with DSD separated by diagnosis and demographic parameters.

Diagnosis	Interviews			Life condition of the participant (n)		
	Total	Age group	n	S	St/E	P
CAH	6	6–13	3	3	-	-
		14–17	1	1	-	-
		18–24	2	1	1	-
TS	5	6–13	1	1	-	-
		14–17	2	2	-	-
		18–24	2	1	1	-
KS	4	14–17	3	3	-	-
		18–24	1	-	1	-
XX-/XY-DSD^1^	4	14–17	2	1	1	-
		18–24	2	-	1	1
Total	**19**			13	5	1

1 including MRKH.

*S* *=* *School.*

*St/E* *=* *Study or education.*

*P* *=* *Profession.*

### Key effects of the training

**Figure 3:**
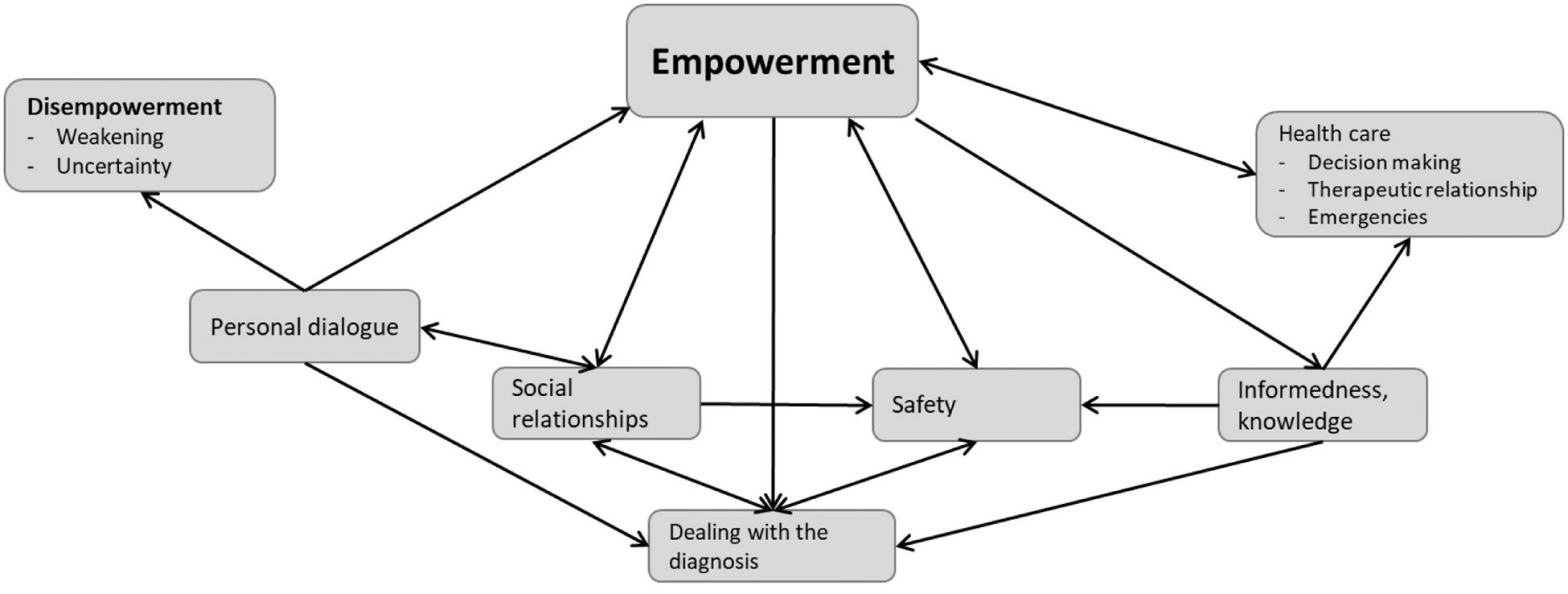
Key effects of participating in training in relation to each other.

Questions regarding HRQoL were not asked directly, but indirectly through the concrete description of life situations. Overall, the training participants predominantly felt empowered and more secure, they described an increase in self-esteem, and their handling of the diagnosis in their respective social environments (family, friends, school) was more open and nuanced. Training participants also felt supported in maintaining their privacy and deciding for themselves who they wanted to tell about their diagnosis. Overall, the training developed a sense of normality so that people with DSD experienced that they were not alone with their diagnosis and that their diagnosis was one of many variations in the development and emergence of human life. The main effects of participation in the training were related to empowerment (Figure 3).

At the same time, it also became clear in the qualitative interviews that the training courses were emotionally upsetting, sometimes also stressful, and required a personal confrontation with the diagnosis. This is not always easy.

“The training also put a lot of emotional strain on me, which of course brought up a lot of things that I had somehow buried. (..) I realized (..) that I hadn't worked through a lot of things, such as my first diagnosis (..) and talking about it did me an incredible amount of good. So (…) it was emotionally upsetting.” (young adult with MRKH)

“And I also found it really great to get to know other parents and [to] see that we actually all have the same worries. (..) Some parents say, like [with] us (..) ‘okay, our child should only find out [about the diagnosis] when it's ready, in puberty’. And then there were the other parents (..) [who] were very open about it from the start (..). It was very interesting. (..) It was a great two days, certainly mentally exhausting (..) but yes, it was really good. (..) And (…) [the trainer] also did a great job, leading and introducing the talks and discussing the topics; I thought that was great.” (mother of child with TS)

### Influence on self-esteem and body image

The frequent reduction in self-esteem and changes in body image caused by DSD-diagnoses, for example due to short stature in TS, lack of pubertal development or menstruation in XX-/XY-DSD, cognitive abnormalities in KS and possible restrictions in the area of fertility and sexuality in all DSD diagnoses, were directly addressed during the training sessions and dealt with by demonstrating coping strategies and developing strengths and resources. The training was perceived as positive and supportive. Participants described the experiences as “empowerment”, 'strengthening', “consolidation”, 'security', 'self-esteem’, even if participation in this 2-day program was not considered sufficient. Rather, it became clear that participation in training can be the beginning of a process that requires ongoing therapeutic and social support. The training courses, in particular the contact with other persons with the same diagnosis, the self-help organizations and the networks established, were mentioned as a valuable basis for this.

“I (..) will never really be one hundred per cent a girl. And (..) because I'll probably have to take hormones for the rest of my life, I'll always have to have it in my head and yes, I don't know how I'll cope with it yet.” (young person with XX/XY DSD)

The desire to have children, sexuality and relationships were reported key issues for people with DSD. While some adolescents and young adults expressed despair about possible challenges in having children, they found conversations about sexuality and relationships in the training courses enriching, but also challenging due to feelings of shame about their own physical limitations. Sharing experiences with others and hearing about the experiences of others was seen as very supportive and helpful. Parents emphasized their uncertainty about how they could best support their children if they had a strong desire to have children and expressed their concerns about their children's well-being in this regard. They also found it difficult to talk to their children about sexuality and possible restrictions. Parents wanted to protect their children, which can lead to conflicts with different needs and also to concealment of the diagnosis (in children). The exchange in training courses was perceived as a relief, especially by parents.

“Other spaces are created [in the training], (..) of course we [the parents] have completely different (..) worries and needs than our son, the way he deals with them. (..) With the other mums and dads (..) I thought (..) it was good that they also explained it again from their point of view, how does the adult father see it? (..) I was then able to relax and let go. (..) So I really found that incredibly pleasant.” (mother of a young adult son with KS)

Diagnosis and medical care outside of specialized centres were often described as traumatic and inadequate. Ignorance and a lack of empathy on the part of practitioners was reported as far too common. As diagnoses are increasingly made prenatally, the connection to specialized centres was perceived as too late, even if from birth on.

All diagnosis groups emphasized that a normal life was possible despite a DSD diagnosis, but called for greater social awareness. Although DSD is more visible in society nowadays, binary gender thinking, narrow social gender role categorizations, taboos and discrimination remain pervasive. Greater education and sensitization are needed both in society and within healthcare systems.

“[When] these people organized themselves [in self-help organizations] and went public, (…) at some point I knew, ‘okay, you're not alone’. Because as a child I always thought I was totally alone. I always thought I was an alien (..) who had been forgotten. Like a leper. And these are things that you hear from many inter [-people], that they have the exact feeling that they (..) have ended up in the wrong world, that they actually belong somewhere else and to end this feeling of loneliness, so to speak, by connecting, [to others with DSD], that is actually a totally great feeling.” (peer counsellor with XX/XY-DSD)

Additionally, peer counsellors reported their impression from their daily praxis that people who had been trained in the Empower-DSD program seemed to be better informed and empowered than those who had never been participating in Empower-DSD. This empowerment also had a positive impact on peer counseling. The peer counsellors themselves experienced their participation in the training as empowering.

Although the training courses required a lot of time and energy from the professionals, they also reported greater fulfilment and satisfaction in their work thanks to improved therapeutic contacts and interdisciplinary collaboration. Particularly with regard to the transition to adult medicine, the training courses can prepare for this phase by strengthening information and empowerment through the promotion of self-initiative and self-responsibility. Early information about the diagnosis, including sensitive issues such as the desire for children and fertility, should avoid taboos and strengthen self-confidence.

“The last training courses in particular were (..) once again really great; (..) with the impression of having really helped people. (..) This project has already built bridges. (..) We regularly (..) had this peer counseling built in, (..) so that you just realized (..) we give each other something.” (physician)

### Evaluation of the training-program by the participants

**Figure 4:**
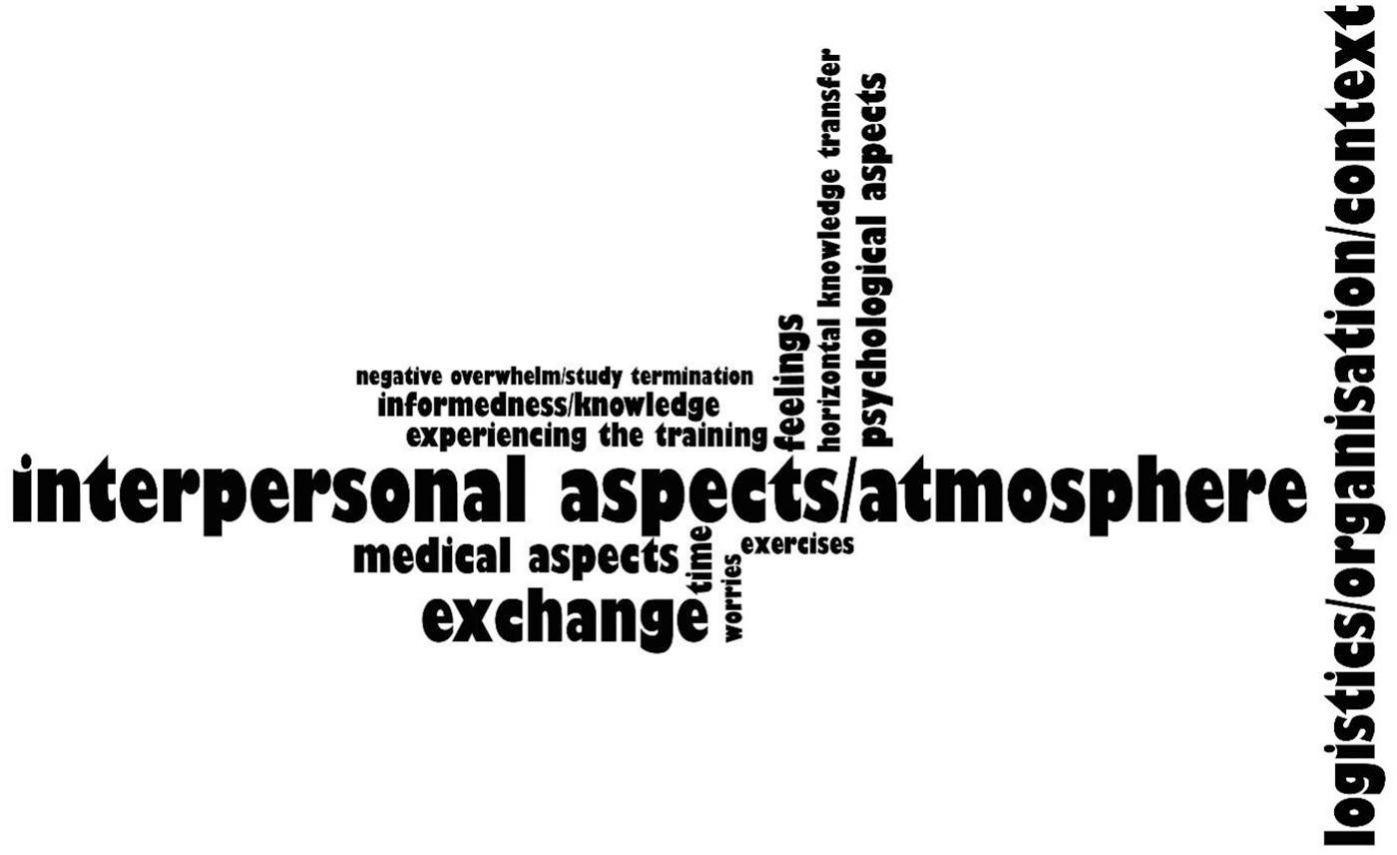
Qualitative evaluation of the most frequent categories on the experience of the training (weighted, font size indicates importance for respondents).

People with DSD (all ages and diagnosis groups) particularly appreciated the expertise of the trainers, the relaxed atmosphere and the responsiveness to questions and problems in the training sessions. The provision of medical information led to a better understanding of the diagnosis and contributed to greater confidence; it was rarely perceived as being too simplistic or too complex. The exercises were seen as a way to lighten the atmosphere. Discussing psychological issues were crucial for some participants, while others found them too emotionally stressful. The division of the training into medical and psychological parts was generally regarded as sensible. The importance of exchanging ideas with other people or families and the need for a good group size and composition for effective training were particularly emphasized (Figure 4). To ensure the best possible experience, groups should be neither too small (at least four participants) nor too diverse, such as those with widely varing age groups, each with different current concerns.

### Triangulation

Although the average HRQoL across all groups did not change, the analysis of individual progression showed that there was a relevant difference from baseline for 174 participants of the Empower-DSD training program after 6 months. It was primarily the groups of adolescents and young adults that showed the highest proportion of demonstrable changes in HRQoL. They showed an improvement in more than 25%, but in 43% also a reduction in HRQoL. The training program encouraged discussion of general, personal and intimate topics that were of great concern to the participants. This resulted in feelings of empowerment and increased self-esteem, and positive experiences of sharing shameful topics and concerns. It also helped them to cope socially with the diagnosis (Figure 4). Dealing with the finality of certain characteristics, whether visible physical features or the impairment of bodily functions, such as infertility, was challenging. Nevertheless, alternatives were discussed during the training, such as different ways of living with a family and coping strategies, including acceptance and less avoidance. This led to an active discussion that was rated positively by the participants. Overall, the training participants felt empowered, their self-esteem increased and their ability to deal with the diagnosis in their respective social environments (family, friends, school) was more open and nuanced (Figure 3). Participants in the training program also felt supported in terms of privacy and in deciding whom to tell about their diagnosis. Their self-confidence was strengthened by answering questions about the diagnosis and practising how to deal with situations in everyday life that caused uncertainty. The two-day Empower-DSD training program was described by some as “emotionally upsetting”, stressful at times, and requiring a personal confrontation with the diagnosis. The need for additional support was underpinned by the striking figures relating to the high proportion of participants with a tendency towards depression (Tables 3, 4). However, this atmosphere and the dialogue between the participants, was highly valued in the analysis (Figure 4). Although open discussion is not always easy, especially during adolescence, the Empower-DSD training program can be seen as the beginning of an important personal dialogue. The need for greater education and awareness in society and within the medical profession should not be underestimated.

## Discussion

### Main findings

The German Empower-DSD project has developed, implemented and evaluated modular training programs for the DSD diagnoses CAH, KS, TS and one for the remaining DSD diagnoses, including chromosomal DSD, MRKH and other XX- and XY-DSDs. For the first time it offers children, adolescents and young adults with DSD and their parents the opportunity to receive structured information about the diagnosis, but also the opportunity to process and cope with it. HRQoL was stable over the whole study population during the time of evaluation, but relevant proportions of participants showed either an improvement or a decline in HRQoL compared to baseline.

### Comparison with other studies

Overall, HRQoL decreased with age and was positively correlated with the participants' HRQoL at baseline. Our baseline examination revealed a reduced HRQoL and well-being in children and adolescents with DSD compared to the general population (16, 25). This highlights the urgent need for psychological support as part of interdisciplinary counseling. The WHO-5 score has been shown to be associated with depression (26). In the Empower-DSD study, already at baseline 28 young adults (50%) with a DSD-diagnosis, 60 parents of a newly diagnosed child (43.2%) and 89 parents of a child with a longstanding diagnosis (36.9%) are suspected of having depression (WHO-5 score <52). The criteria for major depression (WHO-5 score <28) applied to eleven young adults (19.6%), 31 parents of newly diagnosed children (22.3%) and 40 parents of a child with a longstanding diagnosis (16.6%). Multivariable analysis revealed that parents' HRQoL correlated with that of their children at all time points. Therefore, supporting parents is crucial for promoting the individual well-being of their children. The reduced scores in HRQoL could have been influenced by the generally declining HRQoL and mental disorders especially among adolescents during the COVID-19 pandemic (27–29). Anxiety disorders, emotional problems and depression increased during the pandemic (30, 31). As there was no control group in our study, possible interferences cannot be ruled out. Nevertheless, the downward trend in HRQoL during the COVID-19 pandemic was not confirmed across the study participants, who reported benefits such as empowerment, improved self-esteem, and better understanding of their diagnosis.

Interestingly, results at 3 and 6 months after the training also showed that a relevant proportion of adolescents and young adults in particular had a reduced HRQoL. Adolescence and young adulthood are important phases in psychosexual maturation, during which the peer group exerts a significant influence. Coming to terms with one's own body, becoming aware of physical characteristics and dealing with questions about sexuality and infertility can cause insecurity during this period. It is important to continue to offer support after the training in a DSD centre. In the analysis, HRQoL aspects like school and friendships remained low, possibly also due to limited real-life application of strategies during lockdowns. However, after three months, children and adolescents showed improved body image, a trend that continued among young adults after six months. Topics such as shame and body-related concerns, including those relating to the genitals, were openly discussed, which contributed to a sense of normality and community. Participants of all ages and diagnoses expressed high levels of satisfaction (24), which may be due to the open discussion about diagnosis, training's focus on coping strategies, psychological support and peer counseling. Satisfaction with the training programs may have been an indication that the needs of the participants were met. To achieve this, the programs were developed from the outset in collaboration with the relevant stakeholders and self-help organizations for each diagnosis and the content was aligned with the current guidelines on the included DSD-diagnoses (11, 13, 14). The Empower-DSD trainings were delivered in face-to-face group sessions. Some preferred to listen rather than actively participate. Small groups enabled intimate talks, but a minimum size is needed to encourage exchange. It has been shown that group interventions can improve self-esteem and reduce psychological distress (32, 33). The challenge of this training program lay on the one hand in the age-appropriate preparation and teaching of the training content, but also in the heterogeneity of groups, which arose due to the rarity of individual diagnoses, e.g., in the XX-/XY-DSD group. This requires well-qualified trainers. As part of our project, a dedicated DSD Trainer academy was established for this purpose. The ability to respond to individual questions and offer high-quality training was seen by the qualified trainers as a strength.

Interviews revealed that information about DSD often reached families too late. Referral to DSD centres was usually delayed but seen as relieving (34). Evaluating DSD centres—through benchmarking of staff qualifications and care quality—is crucial for ensuring adequate support (35, 36). Most HRQoL studies compared chronically ill children with healthy peers, although it is recommended that data be collected from a diagnostically comparable group to gain knowledge about the normative adaptation process (37).

### Potential limitations

Due to the relatively small number of DSD cases in the participating university hospitals during the planned study period, it was not possible to include a sufficiently large control group of individuals who has not completed the DSD training program. The absence of a control group may have affected the analysis of the quantitative and qualitative data. In the qualitative analysis, the interviewees compared their impressions of working with people who had been trained in the Empower-DSD program with their experiences of working with people before this study period. However, these impressions may be anecdotal and biased. We were able to recruit only about half of the planned children, adolescents and young adults with a DSD diagnosis. The COVID-19 pandemic impacted the preparation of our project, the education of trainers and recruitment due to restrictions such as social distancing and limited access. The shame and fear associated with confronting the diagnosis may be another explanation, given that our participants tended to report lower levels of shame compared to other individuals with DSD in previous studies (24). Consequently, people with higher levels of shame may have been under-represented. While parents of children with DSD were highly willing to participate, adolescents and young adults were often reluctant to join group training. Statistical analysis was limited by the lower-than-expected sample size, rendering some subgroup analyses unfeasible. With regard to imputation of missing values for multivariable analysis of HRQoL in children and adolescents, it cannot be ruled out that the condition “missing at random” was met, as no information was available for the non-responders. Participants who felt the training negatively affected their own or their child's wellbeing may have opted out. In addition, the group of participants was very heterogeneous in terms of diagnoses, phenotypes and health characteristics.

Nevertheless, the content of our innovative study was fully implemented, the training content was developed using a comprehensive participatory approach together with the self-help organizations and the training courses were offered across all participating centres in Germany. Overall, it should be emphasized that the Empower-DSD project had an exploratory approach combined with qualitative analysis and the results for many of the target variables investigated should help to provide a comprehensive view of the situation of families with a child with DSD, but also of the work of professionals and peer counsellors.

## Conclusions

The Empower-DSD project developed a new multidisciplinary standardized training program for children, adolescents and young adults with DSD and their families to complement the continuous care offered in a DSD centre. It provided age-appropriate, interdisciplinary information to empower individuals with DSD in accepting their diagnosis and making informed decisions. The broad involvement of self-help organizations enabled the materials and concepts to be adapted to the needs of the target group. The project has taken important steps and generated knowledge to improve and structure the treatment and care of people with DSD. Participation in the Empower-DSD program can set in motion a process of coming to terms with the diagnosis from both psychological and medical perspectives. With further funding for these programs, the long-term impact on HRQoL, well-being and health, as well as the impact on health care expenditure should be evaluated.

## Data Availability

The raw data supporting the conclusions of this article will be made available by the authors, without undue reservation.
